# Complications of thyroidectomy for large goiter

**DOI:** 10.11604/pamj.2013.16.138.3277

**Published:** 2013-12-11

**Authors:** Toufik Berri, Rachida Houari

**Affiliations:** 1 Department of surgery, Tourabi Boudjemaa Hospital, Bechar, Algeria; 2 Department of surgery, Benzardjab Hospital, Oran, Algeria

**Keywords:** Large goiter, thyroidectomy, morbidity

## Abstract

Thyroidectomy is a routinely common practiced surgery. Morbidity and mortality from thyroid surgery are disregarded nowadays and undervalued in the literature. Perioperative risks and complications still exist for large goiters and can be life-threatening. These complications may occur during the anesthesia and intubation, intra-, or postoperatively. We set out through a case of a large cervical multinodular goiter (MNG) and a review of literature the perioperative complications and how to avoid them. During the total thyroidectomy operation, an accidental devascularisation of a parathyroid gland, a cervical hematoma which was evacuated by surgical reoperation, hemodynamic disorder and a transitory hypoparathyroidism were the postoperative complications that occurred. Surgery for large goiters remains difficult; so adequate preoperative assessment, particular attention and careful operative procedure should be followed to obtain better surgical outcomes.

## Introduction

Large goiters are those which could be identified from a distance [1]. They are commonly reported at females in the fifth decade of life. Large cervical goiters remain a long time asymptomatic, or become early symptomatic by mediastinal compressing signs. Surgery is recommended to avoid this unpredictable evolution and the risk of malignancy. Total thyroidectomy is the procedure of choice, and it is in most cases practiced by the cervical approach [2]. Morbidity rates for large goiters are higher than the rates for smaller goiters [3]. Cases of deaths reported by some authors are mainly due to cardiopulmonary failure. Management of small goiters is standardized for a long time but until now there are few publications detailing how to deal with large goiters. We describe the case of a large MNG, the pre-therapeutic assessment, surgical procedure, the outcome, and finally we emphasize the complications encountered and the risks attached to the surgery for larges goiters.

## Patient and observation

A 50-years-old woman, hypertensive, hospitalized for a large cervical mass appeared 30 years ago. In the family history, her mother, sisters and cousins underwent a surgery for MNG.

**Figure 1:**
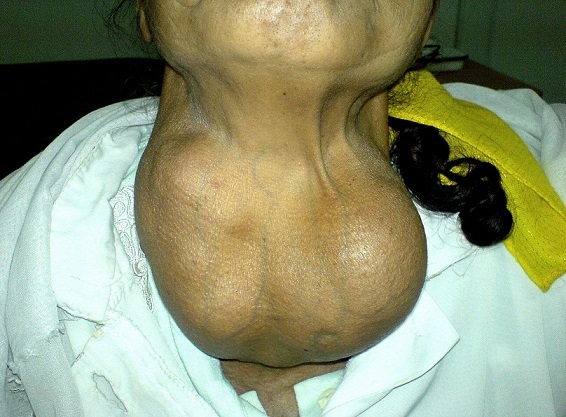
Frontal view of the goiter

Despite of the large volume of the mass, the patient never described signs of cervical compression whatsoever respiratory, digestive, laryngeal, vascular or neurologic signs. She never suffered from thyroid dysfunction. Her neck was deformed by the voluminous formation classified grade III according to the WHO modified classification. The mass took the front and the two sides of the neck. Its surface was embossed and covered by a thin normal skin. There were some veins of the collateral circulation limited to the neck. The goiter measured 18 × 11 cm (Figure 1). The mass was firm, painless, and mobile with the swallowing movements. Lymphadenopathy research was difficult and found no palpable lymph nodes.

**Figure 2:**
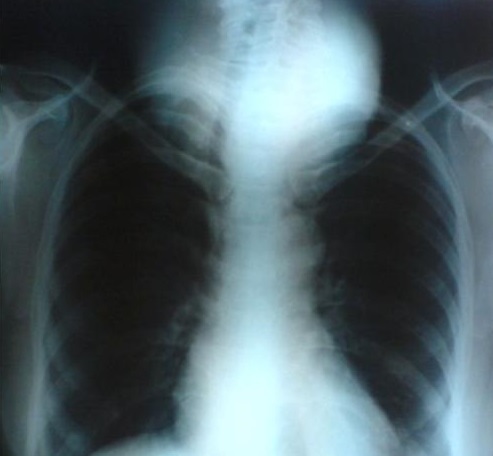
Chest X-ray: the right eccentricity of the trachea

**Figure 3:**
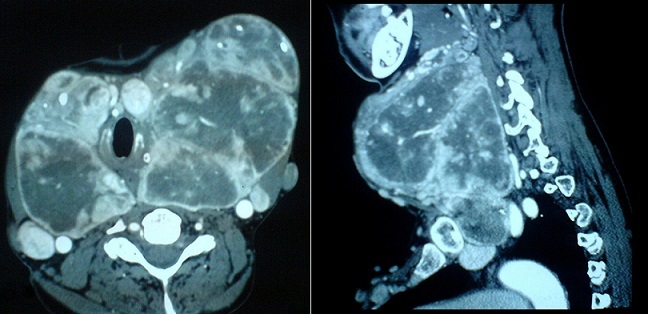
CT scan of the neck

The laboratory tests (T_3_, T_4_ and TSH) were normal. Thoracic radiography showed a large cervical opacity roughly round and strewn with microcalcifications associated with a right eccentricity of the trachea (Figure 2). Cervical and chest CT revealed the presence of a partially calcified thyroid mass slightly plunging in the anterior mediastinum. It took heterogeneously the contrast and then evocate a large MNG. The trachea was surrounded by the goiter, slightly narrowed and right deviated as well as the lower part of the larynx. The right and left vascular axes of the neck (carotid artery and jugular vein) were deviated backward (Figure 3).

**Figure 4:**
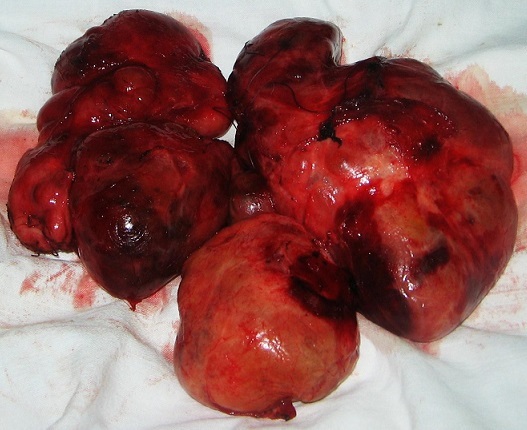
Postoperative specimen

The patient underwent a surgery for her enormous MNG slightly plunging in the mediastinum. Endotracheal intubation was relatively easy by the laryngoscope. The incision performed was a Kocher cervicotomy. There was a multinodular, hypervascularized goiter. Its lower end plunges behind the sternal manubrium. The larynx was deviated towards the right side. The total thyroidectomy was performed in two steps: initially a right lobo-isthmectomy, then the left lobectomy. The retrosternal part of the goiter was released using the finger by the same incision (Figure 4). Both recurrent laryngeal nerves (RLN) were not identified because of the hemorrhage. One parathyroid gland was accidently devascularized and was autotransplanted to the ipsilateral sternocleidomastoid muscle. The operation was finished by double aspiration drainage.

In the first hours after surgery, the patient developed a large cervical hematoma. She was readmitted to the operating room, and after evacuation of the hematoma there was no vessels bleeding. The operation was completed with a double suction drainage.

In the immediate postoperative period, the patient developed hemodynamic collapse requiring the introduction of dobutamine. After 48 hours of hemodynamic support, the blood pressure stabilized and dobutamine was stopped.

Histological study concluded in multinodular colloid goiter. The patient was discharged from the hospital after 20 days in good health.

## Discussion

The management of large goiters is complicated due to the anesthetic or surgical risks that may threaten the life of the patient. These complications can occur pre-, intra-, or postoperatively.

Concerning the anesthesia, the preoperative anesthetic assessment of large goiter is very important to insure a good course of the surgical operation and the outcomes. Cervical and chest CT or magnetic resonance imaging (MRI) are the most reliable methods to identify patient at high risk of perioperative complications. Postoperative complications were predicted by severe symptoms at presentation, tracheal compression of > 50%, pericardial effusion, and a mixed obstructive-restrictive picture on pulmonary function testing [4]. Difficulty of intubation may be caused by an enlarged thyroid gland producing tracheal deviation or compression.

Now most of the major life-threatening complications occur postoperatively [5]. The risk of postoperative cardiorespiratory complications is not negligible; so in the postoperative period of our patient, a cardiovascular collapse occurs mandating the introduction of dobutamine. The etiology of the hemodynamic disorder could not be determined because all the tests were normal (cardiac, thoracic, renal and electrolytes) apart from a slight hypocalcemia and hypophosphatemia.

Concerning the surgical procedure, the complications of thyroidectomy for large goiters still exist although they are rare after standard thyroid surgery. These complications include postoperative hemorrhage, airway obstruction, RLN injury, hypoparathyroidism and thyroid crisis.

The incidence of symptomatic postoperative hemorrhage requiring reintervention amounts to 1.2% [6]. Bleeding occurs in the first hours after surgery. It is manifested postoperatively by respiratory distress, pain, dysphagia and increased blood drainage. The hematoma formed in the neck can lead to tracheal compression, laryngeal edema and respiratory compromise. The patient should be returned to the operating room for evacuating the hematoma and ligation of any bleeding vessels. This complication occurs in our patient three hours after thyroidectomy and the hematoma was evacuated under general anesthesia. There was no active bleeding in the operative field.

Paralysis of the RLN could be transient or seldom permanent [6, 7]. It is the most common complication after thyroidectomy, especially for large goiters, for cancer, or in reoperations. Bilateral RLN injury occurs in 0.4% of total thyroidectomy [6]. Both RLN were not identified in this reported case because of bleeding but there was no palsy of these nerves postoperatively.

A transient or permanent hypoparathyroidism occurs after accidental devascularization of the parathyroid glands during the ligation of the thyroid gland vessels or less commonly by the removal of the parathyroid glands. In this case, we encountered also this kind of incident with an accidental devascularization of a parathyroid gland. We proceeded to its autotransplantation to the sternocleidomastoid muscle. Postoperatively the patient describes a transient hypoparathyroidism signs.

Pneumothorax, pleural effusion, pneumonia, tracheomalacia, laryngeal edema, injury to the esophageal wall, the phrenic nerve paralysis [8] and brachial plexus injuries have also been described. The cervical sympathetic trunk is injured on rare occasions when the goiter extends to the retroesophageal region [7].

For a safe management of patients with large goiters, a complete preoperative assessment, a vigilant surgeon's eyes during all the steps of the excision of the thyroid gland and caution should be observed for the occurrence of early postoperative life-threatening complications.

Most of the postoperative complications would be avoidable if the surgeon has a profound knowledge of anatomy and respects a meticulous procedure of thyroidectomy. The volume of operations contributes to a safe surgical procedure and good outcome [9]. The surgical exposure may be improved by good installation, section of prethyroid muscles and ligation of the middle thyroid veins. Careful and cautious dissection, externalization of the retrosternal part of the gland, and eventually morcellation of the goiter may facilitate the removal of the gland.

The surgeon must take care to practice an adequate hemostasis to avoid postoperative hematoma and the injury of the RLN. Nowadays although, the visual identification of the RLN is the gold standard, the search for the nerve could be difficult by its adhesion to the posterior part of the thyroid or its drive back by the gland. Intraoperative neuromonitoring does not reduce the risk of RLN lesions [10]. To avoid the RLN injury Proye reported basic rules: ligation of the superior thyroid pedicle, ligation of middle thyroid veins, locating the upper parathyroid and recurrent laryngeal nerve at its penetration into the cricothyroid membrane, opening up the thoracic portion of the goiter with the finger, realized lastly. The dissection must be done closer to the thyroid capsule [11].

In order to prevent the external branch of the superior laryngeal nerve during ligation of the superior thyroid pedicle, meticulous dissection of the adventitial tissue between the upper thyroid pole and the laryngeal wall is necessary. To preserve the blood supply of the parathyroid glands, selective ligation of terminal branches of the inferior thyroid artery is required rather the trunk.

## Conclusion

Thyroidectomy for large cervical goiters is a challenging surgical procedure, burdened by high risk of complications. Caution, heightened vigilance and prudent management should be done by the medical team. The pre-therapeutic assessment has a crucial importance for the surgical outcomes.
